# Enigmatic Medicine: a proposed rebranding of emergency medicine

**DOI:** 10.1007/s43678-023-00519-w

**Published:** 2023-05-12

**Authors:** Lee Yung Wong

**Affiliations:** 1 Emergency Department, Austin Hospital, Heidelberg, VIC Australia; 2 School of Business, Law and Entrepreneurship, Swinburne University of Technology, Hawthorn, VIC Australia

**Keywords:** Professional identity, Emergency medicine, Emergency physician, Crowding, Leadership, Satire

## Introductions

*“As a society, emergency medicine represents at the same time our finest suit of clothes and our dirty laundry Now the next generation of emergency physicians will tackle the new problems in emergency medicine, which are in many cases are just modernized versions of the old problems.”* [1]

**Fig. 1:**
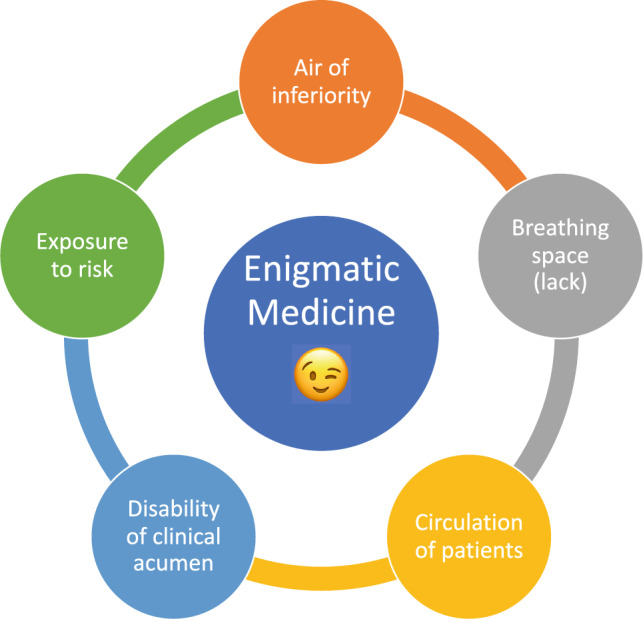
Five tenets of Enigmatic Medicine

Given the state of play of emergency medicine worldwide, with its synonymous problems of crowding [2] and moral distress [3], I propose that an urgent rebranding is needed of emergency medicine and how we self-describe. This is in line with previous calls to examine our professional identity as emergency physicians [4]. A change in name is befitting to suit the change in times, and to reinvigorate the general workforce. Instead of emergency medicine and emergency physicians, we should henceforth be known as *Enigmatic Medicine* and *Enigmatic Physicians*, working in the *Enigmatic Department*. These are our five tenets that encapsulate the enigma of Enigmatic Physicians within the scope of practice of Enigmatic Medicine, and the rationale behind them (refer to Fig. 1).

### Air of inferiority

Despite Enigmatic Physicians’ technical expertise, complex nature of work, and high level of required performance, it is imperative that Enigmatic Physicians and Enigmatic Medicine assume a position of inferiority when compared to other specialities [5]. This is because Enigmatic Physicians are generalists working in a medical culture that esteems specialization, which ultimately breeds values conflicts [6]. Enigmatic Physicians—unlike former emergency physicians—who tend to regard themselves as specialists in resuscitation and critical illness should instead focus on other important matters at work, such as indirect patient care activities of documentation, communication with colleagues and organizational activities. Reassuringly, evidence from two different settings appears to indicate that clinicians in the Enigmatic Department do spend more time in any of these activities than in direct patient care activities of patient communication, diagnostic activities or therapeutic activities [7]. True to form, clinical interruptions such as airway emergencies can be deferred to anesthetics, critical illness managed by intensive care, and diagnostic challenges answered by pathology or radiology departments.

### Breathing space (lack)

Focusing on indirect patient care promotes breathing space in the hectic environment of Enigmatic Medicine. Furthermore, engaging in wellbeing or wellness programs, or consideration for early retirement are mandatory practices to counter the lack of breathing space in the daily work of Enigmatic Medicine. Although bearing some similarity to their counterparts in emergency medicine where Enigmatic Physicians are expected to perform multiple tasks while managing intractable interruptions [8], all individual decisions will be subject to peer review or general scrutiny without taking into account any context or external factors impacting on individual decision-making. If all else fails, increasing one’s non-clinical time or clinical support time is essential to reduce patient contact hours, especially as one increases in seniority. Enigmatic Physicians are strongly encouraged to reduce their patient load despite the sweat, tears and years of brutal training, in order to provide opportunities for the future generation to learn how to circulate patients.

### Circulation of patients

It is important that patients are continually circulated, or ferried, from destination to destination within the *hospital* system, to promote a sense of movement and progression. Hence, all Enigmatic Physicians need to prioritise departing processes for their patients as soon as they meet them. However, it is equally crucial for patients to be held up on departing the Enigmatic Department for two key reasons: confirming the diagnosis or confirming the admitting unit, and additionally confirming that patients are well enough to be admitted to the general wards; these are critical checkpoints in the patient journey. Sick patients who are too well for intensive care, but too sick for the wards should stay in the Enigmatic Department until they deteriorate or improve. Moreover, the time that patients spend in the Enigmatic Department usually helps to provide clarity on both these important steps. It is expected that the decision to admit patients should be heavily contested [6], so Enigmatic Physicians should not be dismayed, but instead focus on performing more tests that will help delineate the admitting unit.

### Disability of clinical acumen

Ordering of tests is important, preferably before the patient is reviewed. This saves time and allows the development of heuristics, which is an essential trait of expert Enigmatic Physicians that has strong connotations with safety and quality. As noted before, test results are critical to inpatient units in order for them to accept the patient, especially to ensure that nothing is missed [9]. Enigmatic Physicians need to be aware that in general, accurate test results take precedence over clinical acumen or physical review. In particular, test results are far more reliable than inconsistent clinician gestalt derived from years of experience, and Enigmatic Physicians are strongly encouraged to prioritise the former.

### Exposure to risk

Universal education and more testing will eradicate instances of missed rare and serious conditions. Any lingering risk is expected to be borne by Enigmatic Physicians, especially risk related to that of the hospital system, as well as those associated with patient discharge and diagnosis [6]. This is crucial, because patients who remain in the Enigmatic Department are their responsibility and not inpatient teams’, despite the theoretical advantages to patient care with the increase in shared responsibility [10]. The risk remains that of the Enigmatic Department alone, and will usually be preserved by the cycle of the circulation of patients back into the Enigmatic Department.

### Rotations and other departmental matters

I also propose that all Enigmatic Medicine trainees undertake some mandatory rotations to ensure their training is rigorous and well-rounded. These include rotations to Intensive Conflict, Infectious Cynicism, Retrieval of Bad Memories in the Middle of the Night and General Melancholy. I also advocate that all Enigmatic Departments are serviced by at least two fully equipped Recrudescence Rooms, a long stay unit, a waiting room large enough to receive patients that are offloaded from ambulances in a timely manner, and, most crucially, a spacious corridor to manage the most critically unwell patients that are unable to be offloaded. The appointment of crowding experts to mitigate crowding will be helpful, but if crowding continues to worsen, I propose that a purpose-built, bigger waiting room should contain the problem.

I hope that these suggestions will not be taken lightly, and look forward to receiving feedback which will, as part of a standard approach to change management, be subjected to working groups and committee reviews before incorporation into policy and protocol, which will be audited in the near future.

## Data Availability

Data sharing not applicable to this article as no datasets were generated or analysed during the current study.
